# Eight Whole-Genome Assemblies of *Yersinia pestis* subsp. *microtus* bv. caucasica Isolated from the Common Vole (*Microtus arvalis*) Plague Focus in Dagestan, Russia

**DOI:** 10.1128/genomeA.00847-17

**Published:** 2017-08-24

**Authors:** Angelina A. Kislichkina, Aleksandr G. Bogun, Lidiya A. Kadnikova, Nadezhda V. Maiskaya, Viktor I. Solomentsev, Mikhail E. Platonov, Svetlana V. Dentovskaya, Andrey P. Anisimov

**Affiliations:** State Research Center for Applied Microbiology and Biotechnology, Obolensk, Russia

## Abstract

We here report the draft genome sequences of 8 *Yersinia pestis* subsp. *microtus* bv. caucasica strains isolated from the East Caucasian (previous name, Dagestan) mountain focus (no. 39), representing the most ancient branch of the 0.PE2 phylogroup circulating in populations of common voles (*Microtus arvalis*).

## GENOME ANNOUNCEMENT

*Yersinia pestis*, the etiologic agent of plague, includes several phylogenetic groups (1). Strains of *Y. pestis* subsp. *pestis* are virulent for a broad spectrum of mammal species and were the causes of the three pandemics (Plague of Justinian, the Black Death, and the Third Plague pandemic). Other endemic strains (*Y. pestis* subsp. *microtus*) circulating in populations of different species of voles are characterized by high virulence to their main natural hosts and laboratory mice; as a rule, however, they were of low virulence or were avirulent for guinea pigs and caused only occasional disease in humans that was not accompanied by outbreaks of human-to-human transmission of infection (2). Our understanding of the population structure, origin, and spread of this major pathogen has increased using whole-genome sequencing to investigate isolates of *Y. pestis*.

The study of distal branches of the plague tree can give additional data for comparative analysis of transformation from the enteropathogenic bacterium *Yersinia pseudotuberculosis* with a fecal-oral route of transmission into hypervirulent vector-borne *Y. pestis* that, as a rule, causes a generalized highly lethal septic infection.

The data of 25-locus multiple-locus variable-number tandem-repeat analysis (MLVA25) typing of *Y. pestis* subsp. *microtus* bv. caucasica strains (3) suggest that the strains from the East Caucasian (previous name, Dagestan) mountain focus (no. 39) represent the most ancient branch of the 0.PE2 phylogroup circulating in populations of common voles (*Microtus arvalis*). To date, only one whole-genome sequence of the strains from this ancient plague focus, which is characterized by a polymorphism of circulating their strains, has been deposited in GenBank (accession number LIYP00000000 [4]). In this study, we sequenced eight additional strains isolated in different years from different parts of this focus.

DNA samples were extracted using conventional SDS lysis and phenol-chloroform extraction methods.

Whole-genome sequencing was performed using the Illumina MiSeq instrument, according to the manufacturer’s instruction. DNA libraries were prepared using a Nextera DNA laboratory preparation kit. The MiSeq reagent kit version 2 was used for sequencing. For each genome, reads were assembled *de novo* using SPAdes version 3.8.1 (http://cab.spbu.ru/software/spades/). Finally, we obtained from 166 to 191 contigs for each genome (Table 1). The genome sizes ranged from 4.50 to 4.57 Mb. Each genome contains 3,927 to 4,215 coding sequences. Each of the strains has two plasmids (pMT and pCD). The pPCP plasmid is absent, as in all other 0.PE2 (*Y. pestis* subsp. *microtus* bv. caucasica) strains. A detailed report of a full comparative genomic analysis will be included in a future publication.

**TABLE 1:** Strain-identifying information and basic statistics on assemblies and annotations

Strain name	Alternative strain name	Raw data accession no.	GenBank assembly accession no.	Size (bp)	No. of contigs	No. of genes		No. of CDSs^a^	
						Total	Coding	Total	Coding
SCPM-O-B-7111	C-746	SRR3501107	MTZY00000000	4,567,036	191	4,466	4,215	4,381	4,215
SCPM-O-B-7005	C-824	SRR3529493	MTZZ00000000	4,569,487	190	4,466	4,214	4,381	4,214
SCPM-O-B-6994	С-739	SRR3529496	MTZX00000000	4,558,616	188	4,451	4,200	4,366	4,200
SCPM-O-B-7042	C-712	SRR3529526	MTZW00000000	4,567,750	182	4,456	4,205	4,371	4,205
SCPM-O-B-7037	C-370	SRR4017164	MIDX00000000	4,558,520	176	4,235	3,985	4,149	3,985
SCPM-O-B-6176	C-535	SRR4017165	MIDY00000000	4,562,336	176	4,234	4,020	4,147	4,020
SCPM-O-B-7040	C-678	SRR4017166	MIDZ00000000	4,564,996	186	4,244	4,012	4,157	4,012
SCPM-O-B-6992	C-700	SRR4017171	MIEA00000000	4,503,787	166	4,164	4,080	3,927	3,927

^a^ CDSs, coding sequences.

### Accession number(s).

The GenBank accession numbers for the eight genome sequences are listed in Table 1.
